# Dentition and Misnomer

**Published:** 1881-10

**Authors:** George N. Monette

**Affiliations:** New Orleans, La.


### DENTITION A MISNOMER.

BY GEORGE N. MONETTE, M.D., NEW ORLEANS, LA.

I have observed with indefinite surprise that the majority of diagnoses made as being the approximate cause of death of the major part of the infants and children in all of our large cities, is that of dentition. I consider this palpably to be a misnomer, and I trust that the term be soon abandoned. How a physiological and natural process could be made pathological and promote death, I can not divine and render consistent; nor can I accuse the Great Architect of being so sadly deficient in point of anticipatory complications incidental to the development of teeth. There seems to be an irresistible and inherent predisposition on the part of the people at large to denominate every infantile disorder as a sequel of the physiological process, dentition, where there are no teeth nor the prospect of any. Each indisposition, in whatever order, and exclusive of extraneous influences, is directly ascribable to dentition, no matter if the infant be only a month old, from the febrile complication to the acute and chronic alimentary derangements, eczema, tinea capitis, atrophia otitis, coughs, etc., so it is said.

When the patient is beyond the dental impeachment then the ever-prolific sagacity of the neighboring *old woman* ventilates the abstruse subject by pronouncing her dictum—worms. These are inherent fallacies, and unless their eradication be complete and speedy, the mantle of ignorant reflection is only wrapped more closely about the medical profession.

Since all infantile diseases are imputed to the teeth, and this being natural and consequent upon development and growth, the unsuspecting parent considers medication unnecessary; and when the physician is called, neglect is the cause remotely of his inability to relieve. Hence the nihil plan of treatment, and a deprecation of our profession, and a fostering of imposters and charlatans.

It has been my experience, and I am quite sure identical with that of all medical men, that all of the former derangements are accredited to the teeth. Climatic transitions are laid aside in the contemplation of the proximate causes, so also improper nutriment, undue exposure from insufficient clothing, hereditary taints—all, indeed, of the ailments are accredited to the teeth, regardless, even, of a pre-existing zymotic origin. Certificates of death are given with dentition as the cause. Such may gratify the people that their child gave up its life for the sake of its teeth, but it conceals the lack of penetration and ignorance of the attendant.

I have been called to hundreds of children with chronic diarrhœa, indeed moribund, simply because that disease was due to dentition.

Let us arouse ourselves from our lethargic couch and cast off the sable mantle that enfolds our panoply with its ignorance. I do not invite discussion, but I can not imbibe such a doctrine, that the simple development of the first principle of nutrition, mastication, or the means of it, should be the paramount provocative of all infantile disorders.—*The Western Medical Reporter.*

